# Breaking the Mould: Comparing 3D-Printed and Composite Bone Models in Orthopaedic Training

**DOI:** 10.7759/cureus.75520

**Published:** 2024-12-11

**Authors:** Charlotte Binnie, Yumna Nayab, Christopher Bano, Leo Gundle, Jerome Davidson

**Affiliations:** 1 Plastic Surgery, St George's Hospital, London, GBR; 2 Trauma and Orthopaedics, Guy's and St Thomas' Trust, London, GBR; 3 Plastic Surgery, Guy's and St Thomas' Trust, London, GBR

**Keywords:** 3 dimensional printing, distal end radius plating, orthopaedic registrar training, simulation in medical education, simulation models

## Abstract

Background and aim

Synthetic composite bone models (reinforced solid foam) have become the standardised material used in practical orthopaedic education. However, with discussions regarding whether composite foam truly replicates human bone, there has been a drive to explore other available models. Three-dimensional (3D) printing has risen in both popularity and availability, providing a new option in the creation of anatomically accurate bone models. We designed a pilot study to assess whether a new formulation of synthetic bone provides the same tactile feedback that is essential for training purposes.

Method

Orthopaedic trainees of various grades across two London hospital trusts were invited to participate in a distal radius fixation workshop. As part of the workshop, trainees were asked to complete the following three tasks on the two different models: Kirschner-wire driving, pilot hole drilling and screw insertion. Participants were blinded in this trial and not informed which model was made via 3D printing or the conventional composite bone. Following completion, participants provided feedback on tactile feedback for each task on each model.

Results

Twenty-three orthopaedic trainees participated in the workshop, with overall majority agreement in all clinical skills that the 3D-printed model provided better tactile feedback. Three-dimensional models were rated superior in K-wire driving (mean score 7.39 vs 4.82; p<0.001) and pilot hole drilling (7.87 vs 4.96; p<0.001), with no significant difference in screw insertion. Qualitative feedback from testers noted a more anatomical representation of the 3D-printed bone, in addition to an overall better representation of the corticomedullary junction.

Conclusion

Overall, 3D printed models provide a new high-fidelity and sustainable option when seeking bone models for modern-day orthopaedic training. At present composite bone remains the standard for workshops. However, with the growing availability of 3D-printing models, and as supported by this study, they crucially provide the medium for future orthopaedic surgeons to learn and gain confidence.

## Introduction

Anatomical models form an essential part of surgical education, not only to aid anatomical learning but also to provide life-like tactile input in procedure simulation of the transition from hard to sort bone. Specifically in orthopaedic training, bone models are integral in the early development of the psychomotor skills required for fracture fixation [1]. With increasing pressures for operating room efficiency, restrictions on work hours and a reduction in theatre time for trainees during the COVID-19 pandemic, the use of simulation training can be beneficial for orthopaedic surgical trainees [2,3].

Composite bone models have become the most widely employed material for orthopaedic education due to their anatomical and physical similarities to human bone [4,5]. These models consist of an outer layer of epoxy resin surrounding a foam core to represent the cortical and intramedullary structure of bones [4]. These can be procured from multiple companies but have been colloquially named "sawbone," a term originating from one company that registered the name "Sawbones" [6]. Also, although the manufactured architecture of composite bone replicates human bone, there is a varying degree of evidence in support of its life-like characteristics [7].

Recently, there has been an emergence in popularity for the three-dimensional (3D) printing of bone models. These models can also be designed to match human bone anatomically and can incorporate other materials to replicate tactile feedback [6]. Similarly, a liquid resin is placed into the 3D printer, and based on the input design, it hardens into plastic to mimic the cortical component of bone. Specifically, in the Claribone model, the printed shell is filled with Plaster of Paris to represent the medulla, thus reproducing the corticomedullary junction essential for replicating tactile feedback [8]. Interestingly, "Claribone Ltd, United Kingdom" uses a plastics uplifting firm that uses refuse plastic from products as building material, to avoid landfill waste [8].

Both forms of model utilise different methods and materials to imitate the corticomedullary interface. This junction provides the tactile feedback essential for developing basic surgical techniques of Kirschner-wire driving, pilot hole drilling and screw insertion in orthopaedic workshops [5,9]. Simulation training not only provides a safe opportunity for trainees to develop these skills, but with service restrictions and increased trainee numbers, it provides more opportunity to complete the procedure [9]. Specifically, distal radius fracture fixation is a procedure often completed by junior orthopaedic trainees, which can encompass the skills listed above, and therefore is an ideal anatomical basis for trainee workshops.

With this in mind, the primary purpose of this study was to establish whether our 3D-printed model offered a viable alternative for orthopaedic training to composite bone models in the three skills previously outlined, with a secondary objective of establishing whether this workshop helped trainees gain confidence with distal radius fixation.

## Materials and methods

Distal radius models, both composite and 3D printed, were trialled by orthopaedic surgeons (23 in total) at various stages of training across three London hospital sites over two trusts (Guy's and St Thomas' NHS Foundation Trust and St George’s University Hospitals NHS Trust).

**Figure 1:**
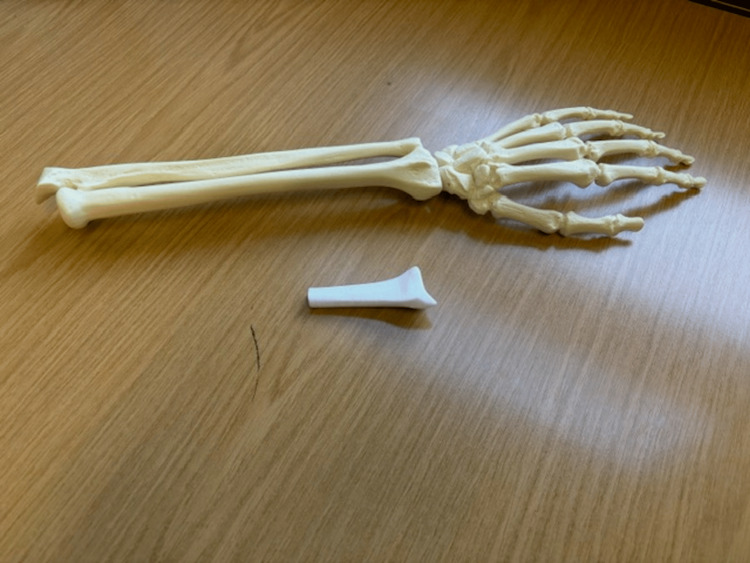
Pictures of model A (composite bone model) above, and model B (3D-printed bone model) below.

Two types of bone models were sourced for the skills lab as follows: a complete forearm composite bone model (model A) and a 3D-printed simulator of the distal radius (model B), demonstrated below in Figure 1. Model A was procured via the "Sawbones" company - a forearm and hand bone model made from an outer epoxy resin and inner foam core [4,6]. Model B was sourced from "Claribone" who uniquely designed the template for 3D printing of the distal 7 cm of the human radius from an outer hardened liquid resin with an inner core of Plaster of Paris [8]. We acquired four models of A and six of B for use in this study, providing ample surface area for fixation practice. The timeline from request to resource attainment for both models was less than a week.

Participants were recruited through word of mouth and invitation on the departmental messaging platforms, with inclusion criteria requiring current practice in an orthopaedic rotation. All participants provided verbal and written informed consent for voluntary participation in this project, evidence of all consent alongside final questionnaires were kept anonymously for record. The NHS Research Ethics Committee Tool was consulted which suggested ethical approval was not required for this project [10]. Therefore, we did not present it to an ethics committee for review.

**Figure 2:**
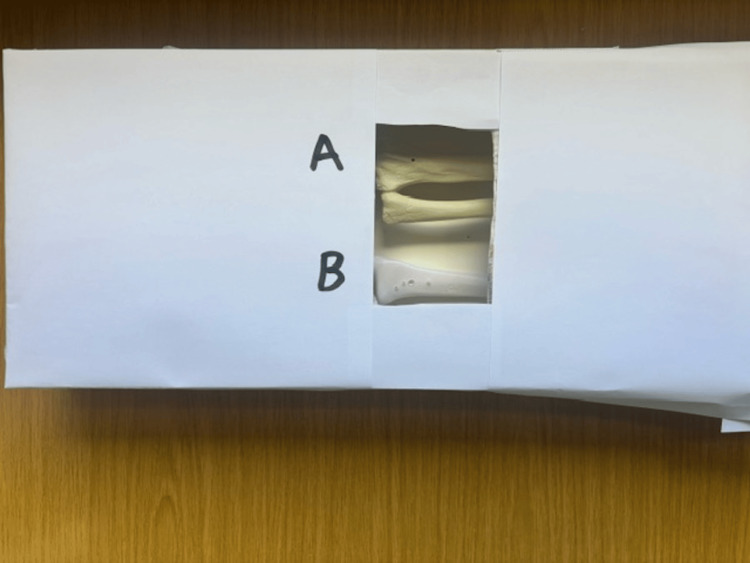
Image of cloaking device used to single blind participants.

Surgeons were invited to complete Kirschner-wire driving, pilot hole drilling and screw insertion on each model. These tasks were chosen on the basis of essential basic orthopaedic skills for all fracture fixation, and so key to developing and maintaining as orthopaedic trainees. As a blind trial, participants were not informed prior to the skills lab which simulator was the 3D-printed model and which was the composite bone model. Instead, these models were labelled A (composite bone) and B (3D-printed model). The models were isolated within a box with an opening to allow for the completion of the procedure without obvious visual clues influencing results (Figure 2). Each participant completed each task sequentially in the same order - Kirschner-wire driving, drilling pilot holes and screw insertion. This allowed for replication and cross-comparison of results, with an initial sequence designed to allow for ease and repeatability of workshop provision across trusts. Following the session, each surgeon was invited to complete a questionnaire acquiring tactile feedback rating for each skill and its likeness to real bone. This was rated on a 10-point Likert scale (with 10 being optimal tactile feedback). Participants were asked to provide further feedback on which model they believed would be more appropriate for training as well as providing their qualitative impressions. All numerical results and qualitative feedback for the workshop were anonymously incorporated into this study. Data were analysed using Microsoft Excel (Redmond, WA: Microsoft Corp.).

Surgeons were split into the following three stages of training: pre-registrar (foundation year one to core trainee year two), junior registrars (speciality trainee year three to five) and senior registrar (speciality trainee year six to eight).

## Results

Twenty-three participants took part in this study, consisting of 60.9% (14) pre-registrar, 21.7% (five) junior registrars and 17.4% (four) senior registrars. Data were collected on participant experience, confidence levels and qualitative feedback on the two models, with a specific comparison of tactile feedback during the following three tasks: driving K-wires, drilling pilot holes and screw insertion (Table 1).

**Table 1:** Demographic breakdown of participants in this pilot study.

Demographics	Values		
Grade	Pre-registrar - 14 (60.9%)	Junior registrar - 5 (21.7%)	Senior registrar - 4 (17.4%)
Trust	GSTT - 17 (73.9%)	St George’s - 6 (26.1%)	
Confidence in completion/assistance of distal radius fixation	Yes - 17 (73.9%)	No - 6 (26.1%)	

This study demonstrated a statistically significant difference in tactile feedback scores between model A and model B for two out of three tasks. For driving K-wires, model B had significantly higher scores compared to model A (t= -6.31, p<0.001). Similarly for drilling pilot holes, model B proved superior to model A (t= -7.73, p<0.001). However, for screw insertion there was no statistical significance between the two models (t=01.60, p=0.123), suggesting that both models were comparable in this task. Despite a relatively smaller sample size, the extrapolation of results is not on face value but backed by statistical analysis as demonstrated in Table 2. These results overall suggest the superior tactile feedback and realism of model B, highlighting its potential use as a more realistic training tool in the training of orthopaedic surgeons. 

**Table 2:** Mean scores, standard deviation (SD) and confidence intervals for each model isolated for each task.

Task	Model	Mean (SD)	Lower CI	Upper CI
Driving K-wires	Model A	4.82 (0.57)	4.13	5.52
	Model B	7.39 (0.61)	6.78	7.99
Drilling pilot holes	Model A	4.96 (0.63)	4.32	5.59
	Model B	7.87 (0.58)	7.39	8.35
Screw insertion	Model A	6.09 (0.74)	5.17	7.01
	Model B	7.00 (0.79)	6.30	7.70

Additionally, we asked participants to provide qualitative feedback on their experience, comparing practice with the two different designs of models. This was done using the following open-ended question: "Please provide feedback on your experience comparing practice with the two different designs of the model." A summary of the results is demonstrated in Table 3. Overall, on reflection, multiple participants stated that model B (3D-printed model) was "more realistic," specifically commenting on characteristics such as the "softer medulla" and the bicortical nature. Feedback regarding screw insertion reported model A to be superior in comparison as there was more engagement with the thread, whilst in model B participants struggled to sink the screw due to the nature of external material.

**Table 3:** Summary of quantitative data answering the question: “Please provide feedback on your experience comparing practice with the two different designs of the model.”

Theme	Description	Example quotes
Realism and tactile feedback	Model B was praised for its realistic feedback and representation of the medulla and cortex	“Model B much more realistic. The bicortical nature of the model much better represents real life.”
Anatomical accuracy	Model B was described as more accurate in simulating real bone structures	“Anatomically more accurate with model B. Increased resistance with model A and less tactile feedback.”
Task-specific feedback	Model A was occasionally preferred for screw insertion due to better thread engagement	“Model A was good for tactile feedback with screwing but not realistic for K-wires or drilling”
Challenges	Participants noticed difficulties with screw insertion in model B due to material properties	“Struggled to sink the screw due to the nature of the external material in model B”

## Discussion

Orthopaedic surgical training heavily relies on hands-on skills and practice to develop technical skills and improve surgical technique and confidence. Composite bone models have been used traditionally in simulation training, replacing the previous preference for cadaveric model teaching [5,11]. However, with the emergence of 3D-printed models, we hypothesised whether this could become the next viable alternative for orthopaedic training [12]. Despite being around for 40 years, the practical utilisation of 3D printing is newly diversifying within the orthopaedic field [13]. Currently, 3D printing with computer-assisted design aids orthopaedic surgeons with pre-operative planning, surgical guides, personalised implants and customised prostheses; however, it is yet to be incorporated as an adjunct for training [14].

When comparing the costs in the procurement of the different models, it was noted that composite bone models of the forearm bones were priced at around £45, whilst the 3D models from the company "Claribone" were £10 per distal radius [7,15]. Pricing for 3D-printed bone can vary widely depending on material choice, size of model and customisation. Specifically, in this study, the 3D-printed model was significantly shorter, measuring only 7 cm in length as a pure distal radius model, which likely influenced the final cost. Additionally, this 3D model from "Claribone" utilises recycled plastics which again can influence the cost. Not only can the material and size influence costing but it can also affect the resultant tactile feedback and so limit the extrapolation of results for all 3D models, thus supporting the need for further larger-scale trials.

The study followed a single-blind protocol by isolating the anonymised models within a cloaking device. However, in practice, this was difficult to fully orchestrate, particularly with more experienced surgeons being more familiar with the structure of the composite bone model, thus limiting the ability to completely blind participants and so has the potential to skew results.

Devices sourced for completion of workshop activities were, in fact, building tools to replicate orthopaedic surgical devices such as wire drivers and cancellous screws. We utilised power drills, short wood screws and cross-head screwdrivers. All devices used in the workshop were able to imitate equipment used intra-operatively and so provide the appropriate tactile feedback, but due to slight differences, there remains some disparity in the likeness of the simulation training.

In regard to the demographics of this study, the majority of participants (60.8% {14}) were in the pre-registrar stage of training. This is beneficial for future research beyond this pilot study as this group of trainees are at the early stages of orthopaedic learning and so ideal candidates for simulation training. Additionally, as previously referenced, more senior surgeons recognised the composite bone models despite blinding, and so by being a smaller proportion or participants, this limits the influence of final data. However, junior staff also has less experience assisting and completing distal radius fixation on patients and thus has less experience with the true tactile feedback of human bone. Specifically, in this data set, roughly half of the participants had assisted or completed less than five distal radius fixations, demonstrating the lack of experience but the necessity for simulation training. Therefore, another limiting factor is the interpretation of a true human representation of the tactile feedback of the models. Additionally, only 23 participants consented to be included in this study, and thus, this small sample size limits the extrapolation of results. A further limitation of this study is the limited amount of prior research available on this topic, which constrained the scope of our literature review. As interest and research in this area continue to grow, we hope to expand and elaborate on this aspect in the future.

Data were collected from orthopaedic trainees affiliated with two different trusts in London, United Kingdom. Although only 23 trainees took part, gathering feedback from two different hospital sites with distinct training pathways helps support the extrapolation of results for potential application by other hospitals.

Electronic questionnaires were designed to gather nominal data representing the degree of tactile feedback on a linear scale to allow for comparison of models. Although tactile feedback is subjective to each person, ensuring that each task was completed sequentially on both models allows for a direct comparison, enabling the subjective degree of difference to be applied to each model. Data interpretation using t-testing was performed on the two samples to calculate confidence intervals and p-values, allowing for statistically supported conclusions and thus supporting the final conclusions of this pilot study, with results defined and recognised as significant at a p-value of less than 0.001.

## Conclusions

Overall, this study demonstrated that 3D-printed bone models provide a superior tactile experience compared to composite models in the simulation of pilot hole drilling and Kirschner-wire driving for distal radius fixation. The anatomical accuracy and realistic representation of the cortico-medullary junction in the 3D model offer new advantages as an adjunct for the development of essential orthopaedic skills. However, as a novel method of manufacture, 3D printing accessibility is limited, and whilst growing in popularity to become a viable option, this study also supports the benefits of composite bone models in simulation training.

As a pilot study, this represents the first step in developing support for new options for bone models. Further large-scale research is required to validate the use of 3D-printed models in different orthopaedic training scenarios. Future studies should aim to involve a greater number and variety of orthopaedic trainees trialling models of different designs and materials to help establish the optimal form of orthopaedic training device. Furthermore, with a further drive for more sustainable and innovative tools in training, this study represents the potential to revolutionise orthopaedic education for the next generation of surgeons.
